# Statistical points and pitfalls - series - introduction

**DOI:** 10.1007/s40037-015-0247-z

**Published:** 2016-01-19

**Authors:** Jimmie Leppink, Kal Winston, Patricia O’Sullivan

**Affiliations:** Maastricht University, Maastricht, The Netherlands; University of Liverpool Online, Amsterdam, The Netherlands; University of California, San Francisco, USA

The overall purpose of the ‘Statistical Points and Pitfalls’ series is to help readers and researchers alike increase awareness of how to use statistics and why/how we fall into inappropriate choices or interpretations. We hope to help readers understand common misconceptions and give clear guidance on how to avoid common pitfalls by offering simple tips to improve your reporting of quantitative research findings. Each entry discusses a commonly encountered inappropriate practice and alternatives from a pragmatic perspective with no mathematics involved. We encourage readers to share comments on or suggestions for this section on Twitter, using the hashtag: #mededstats.

## To test or not to test, that is the question

Research may result in qualitative and/or quantitative data. For each approach, there are numerous questions about handling data, such as how to present data or key findings. We focus in this entry on a question that is of crucial importance in all research that includes a quantitative component and yet is frequently ignored or forgotten: ‘to test or not to test?’ In other words, given your particular situation, when is it appropriate to do a statistical test?

The answer to the core question of this entry—whether or not to engage in hypothesis testing in a study at hand—has three facets, which can be summarized in three sub-questions:

- Do any of the research questions call for a hypothesis test?
- Do the data at hand allow for the use of a hypothesis test?
- Does a hypothesis test have meaning given the observed magnitude of a relation of interest?

In the following, we explain why the use of hypothesis tests is defendable only when each of these sub-questions can be answered with a clear ‘yes’, and that in any other case we should limit ourselves to numerically and graphically summarizing descriptive statistics (e.g., means, standard deviations, correlation coefficients).

### Do any of the research questions call for a hypothesis test?

Firstly, and probably most importantly, there is no use in hypothesis testing if none of the research questions we wish to address call for a hypothesis test. That is, if we have no research question about a phenomenon of interest beyond the specific subjects (e.g., individuals or centres) under investigation, hypothesis testing has no use. Hypothesis tests have been designed to generalize the findings from a sample at hand to a population from which the sample has been taken. For instance, assessment of students enrolled in a course in a particular programme has the goal to measure knowledge and/or skills on the part of these specific students; there is no need to apply any statistical test, for we are not generalizing to other students. However, suppose we have a sample of medical students from the United Kingdom perform a novel form of knowledge progress test and we wish to generalize the findings to all medical students in the United Kingdom, the use of statistical tests may make sense if the conditions outlined in the following are satisfied. In other words, whenever we have access to full population data (e.g., all the students in your course), numerically and graphically summarizing descriptive statistics is all we need to do. The same holds when we are dealing with a very particular sample of individuals (e.g., from a particular centre) and have no intention to, and probably also better not, generalize the findings to a broader population (e.g., to very different centers).

### Do the data at hand allow for the use of a hypothesis test?

If one or more research questions in our study call for a hypothesis test, the next question we face is whether the data at hand allows for the use of a hypothesis test. We will focus on two rather common sample requirements in this entry: representativeness and size.

#### If the sample is not representative of the population, do not perform hypothesis tests

If the aforementioned sample of medical students consists of top-motivated students only, then the sample is not representative of all medical students and generalizing the findings to all medical students in the United Kingdom may make little sense. A more sensible approach is to summarize and plot key findings and recommend suggestions for future research that extends beyond the very specific context of the current sample. If we want to generalize our findings, then we need a sample that is representative of the population of interest; random sampling—drawing your sample such that the laws of probability apply—is considered one approach to obtaining such a sample [1].

#### In very small or very large samples, hypothesis tests have little to no value

Hypothesis testing is based on the idea that the sample at hand is much smaller than the population we wish to generalize to. The population should be at least ten times the size of the sample [2]. In cases when a sample makes up a larger proportion of the population, the uncertainty around sample-based estimates is exaggerated and test outcomes will be biased. For instance, if our sample comprises 50 of the 60 individuals (83 %) from a particular population, it makes more sense to summarize the findings of the 50 individuals than to use hypothesis testing to generalize to the ten other individuals who are missing.

Additionally, as a rule of thumb, very large samples (say, a few hundred observations) are generally powerful enough to detect statistically significant relations of any magnitude, however small and practically insignificant. Consequently, the relevance of the finding of a hypothesis test is arguable. With large samples, visual inspection of summaries and graphs of the data can allow a researcher to summarize the situation regardless of whether a hypothesis test is used. At the same time, when a sample consists of only a few—say fewer than ten—observations, even relations that appear strong by straightforward inspection of the data may not pass a statistical test. In short, very small samples are not powerful enough even for strong relations while very large samples may detect even small relations that arguably have no importance to the field.

### Does a hypothesis test have meaning given the observed magnitude of relations of interest?

The question of practical significance or meaning of sample findings to the field is of crucial importance in medical education. Even if a research question and the data at hand allow for the use of a hypothesis test, the question remains how meaningful an observed relation of interest is to the field, given its magnitude. If that observed relation is so close to zero that it has hardly any implications for the field, a hypothesis test does not add anything. For example, a massively expensive intervention increasing student exam performance by 1 %, or the fact that female students perform, on average, 0.5 % better than male students, may not have a lot of meaning to the field even if a statistical test yields a significant outcome. Therefore, we suggest refraining from conducting a statistical test or reporting the *p*-value that may accompany the descriptive statistic.

### To conclude: when to test and what to do in any case

Hypothesis testing may have a use when (1) a particular research question calls for a hypothesis test, (2) the data at hand allow for the use of such a test, and (3) the magnitude of a relation of interest is such (i.e., neither very small nor very large) that we are still not sure how meaningful it is for medical education practice. However, even in these cases, hypothesis tests should be treated as advisors rather than as supervisors. Authors should acknowledge the limitations of a statistical test and accurately describe the context in which a study at hand has taken place, to describe the outcomes of the study, provide suggestions for future studies, and leave to readers to decide to what extent findings may be of use in their context. Finally, a good numerical and graphical representation of descriptive statistics is in any case more meaningful than testing hypotheses, especially where the latter is superfluous.
